# Chloroform in Cholera

**Published:** 1849-01

**Authors:** 


*Chloroform in Cholera.*—Mrs. Smith, aged fifty-five, of bilious temperment, prone to diarrhœa, was on Monday last seized with all the symptoms of an attack of English cholera—vomiting, purging, excessive spasm of body and extremities, small rapid pulse, and insatiable thirst. The dejections were of a frothy character, resembling yeast, and the matter rejected from the stomach of the same nature. Recourse was had to the usual remedies prescribed in this disease, with strong terebinthinate stupes to the abdomen and calves of the legs, and injunctions given as to diet, &c. Having attended her before for like symptoms, I prognosticated a favorable and speedy result. About three o'clock on the following morning, I was hastily aroused by her husband, as the patient had become much worse. All her symptoms had increased to an alarming degree; the spasm was universal and excessively violent, "as if knots were being tied in her bowels;" vomiting incessant; countenance livid and cold; articulation feeble, praying to be released from her sufferings. As all the medicines had been rejected, I thought it fruitless to continue them, but at once decided upon administering chloroform. A mixture composed of the following was prescribed:—

℞. Chloroform, gt. xvi; aquæ vitæ (cogn.), ℥j.; aquæ destil., ad ℥vj. M.

A forth part was given immediately, which had a partial but most satisfactory effect: an abatement of all her symptoms was the immediate consequence. In two hours a disposition to recurrence manifested itself, when a second dose of the mixture was administered, which entirely controlled all spasms, vomiting, and purging. She expressed herself "very comfortable," and fell into a quiet sleep. At nine o'clock I again saw her, and found her suffering only from some febrile symptoms, accompanied with much exhaustion. She was ordered cold rice and mucilaginous drinks, and had the chalk mixture with nitric ether prescribed. A dose of ox-gall (gr. x.) was given in the course of the day, which produced three bilious evacuations and some disposition to vomiting, which soon passed away. In two days she was declared convalesent. In 1832, when the cholera visited this place, my patient was attacked, but she declares her sufferings then were nothing in comparison with her late disorder. The two remaining doses of the chloroform mixture were ordered to be carefully preserved in case she had any return of her symptoms. A daughter, grown up, who had assiduously attended upon her mother, was on Wednesday evening seized in precisely a like manner, except that the dejections were more abundant and frequent; and the mother, without hesitation or appeal for advice, gave her the two remaining doses of the mixture. The same magic result followed; the first dose was only partial in its effect, but the second completely subdued the disease. When I called on Thursday, the gratifying anouncement was made to me of the success of my medicine in a second case.

Perhaps I am not justified in calling these decided cases of Asiatic cholera, but the disease in its latter stage, in the case of the mother, assumed a much more severe type than our English form usually bears.

Without offering any remarks upon the *fons et origo* of the malady in its worst form, and with prospective fears for its soon visiting our shores, I am but too happy (in conjunction with Mr. Brady) in being able to report so favora ly of a remedy which I believe only requires to be more extensively tested to be appreciated.—*London Med. Times.*

